# Reversible Metabolic and Liver Disease in Complex III Deficiency: Novel Variants Expand the Reported *UQCRC2*-Associated Phenotype

**DOI:** 10.3390/cells15070596

**Published:** 2026-03-27

**Authors:** Graeme Preston, Ibrahim Shammas, Filippo Pinto e Vairo, Anna Ligezka, Carlos Alberto de Moura Aschoff, Fabiano Poswar, Ida Vanessa D. Schwartz, Tamas Kozicz, Eva Morava

**Affiliations:** 1 Department of Clinical Genomics, Mayo Clinic, 200 1st St. SW, Rochester, MN 55905, USA; graeme.preston@mssm.edu (G.P.); dr.ibrahim.shammas@gmail.com (I.S.); eva.morava@mssm.edu (E.M.); 2 Department of Genetics and Genomic Sciences, Icahn School of Medicine at Mount Sinai, 1425 Madison Ave, New York, NY 10029, USA; 3 Department of Medicine, Division of Gastroenterology and Hepatology, Mayo Clinic, 200 1st St. SW, Rochester, MN 55905, USA; 4 Post Graduate Program in Genetics and Molecular Biology, Federal University of Rio Grande do Sul, Avenida Osvaldo Aranha, 338, Porto Alegre 90040-060, RS, Brazil; 5 Medical Genetics Service, Hospital de Clinicas de Porto Alegre, Rua Ramiro Barcelos 2350, Porto Alegre 90035-003, RS, Brazil; 6 Department of Anatomy, University of Pecs Medical School, Pecs Szigeti út, 12 7624 Pecs, Hungary; 7 Department of Biophysics, University of Pecs Medical School, Pecs Szigeti út, 12 7624 Pecs, Hungary

**Keywords:** OXPHOS, mitochondrial, complex III, Seahorse, hyperammonemia, microdeletion 16p12.2

## Abstract

**Highlights:**

**What are the main findings?**

- Biallelic pathogenic variants in *UQCRC2* cause mitochondrial complex III deficiency with recurrent metabolic crises, neurologic dysfunction, and variable clinical severity.
- Liver dysfunction, encephalopathy, and complex III abnormalities are common but often reversible, with potential for full recovery.

**What are the implication of the main findings?**

- Patients with 16p12.2 microdeletion and acute metabolic decompensation should be evaluated for pathogenic *UQCRC2* variants *in trans* and for mitochondrial disease.
- These data highlight the importance of consistent respiratory and biochemical assessment of individuals with *UQCRC2* variants to support continued understanding of the *UQCRC2*-associated mitochondrial disease phenotype.

**Abstract:**

Introduction: Ubiquinol–cytochrome c reductase core protein II (*UQCRC2*) encodes a core subunit of the mitochondrial electron transport chain (ETC) complex III (CIII). Biallelic pathogenic variants in *UQCRC2* have been associated with mitochondrial disease characterized by lactic acidosis, developmental delay, hepatopathy, and episodic metabolic decompensation. Methods: We reviewed the biochemical phenotypes of 14 individuals possessing *UQCRC2* variants, including two novel cases. We performed biochemical studies of mitochondrial respiration and oxidative phosphorylation (OXPHOS) complex measurements in patient-derived fibroblasts. Results: We report reduced CIII activity in a majority of individuals possessing variants in *UQCRC2*, as well as biochemical findings consistent with impaired mitochondrial energy metabolism, though impairments in mitochondrial respiration were variable. The two previously unreported, unrelated patients possessing the likely pathogenic missense variant c.361T>C, p.Tyr121His in *UQCRC2 in trans* with a 16p12.2 microdeletion encompassing *UQCRC2* showed milder phenotypes, less severe metabolic decompensations, and no long-term neurological impairments. Both individuals display reduced CIII activity and mitochondrial respiratory dysfunction. Discussion: These data expand the current understanding of genotypes associated with *UQCRC2*-associated mitochondrial disease to include the novel 16p12.2 microdeletion. These data also highlight the consistent biochemical phenotype associated with *UQCRC2*-associated mitochondrial disease, and the need for consistent biochemical and respiratory assessment of individuals possessing *UQCRC2* variants to further our understanding of this phenotype.

## 1. Introduction

The mitochondrial respiratory chain, comprised of four enzyme complexes, is responsible for cellular energy production [1]. Complex III (CIII) consists of eleven structural subunits, predominantly encoded by nuclear genes, with the exception of the main structural subunit, cytochrome B (MT-CYB), which is encoded by mitochondrial DNA [2]. Although CIII-related mitochondrial diseases are rare, they still constitute 7–10% of all mitochondrial disorders [3]. One of the CIII subunits, *UQCRC2*, is a nuclear-encoded CIII subunit associated with a unique mitochondrial disease. To date, only 12 patients have been documented in the literature with pathogenic *UQCRC2* variants [4,5,6,7,8]. The typical presentation of this autosomal recessive disorder is recurrent acute metabolic decompensation with features consistent with malfunction in the respiratory chain, lactic acidosis, liver dysfunction with potential severe hyperammonemia, and hypoglycemia responsive to glucose infusion [4]. Unfortunately, several patients were reported with acute liver dysfunction resulting in death, as well as neurological complications including seizures, developmental delay, recurrent encephalopathy, and, rarely, Leigh syndrome [4,7]. Neither the effect of early treatment nor the long-term outcome of patients has been previously reviewed.

Notably, the 16p12.2 region possessing the *UQCRC2* gene is highly repetitive and displays genetic instability. The recurring microdeletion at 16p12.2, including *UQCRC2*, can present with heterogeneous clinical features, including developmental delay, intellectual disability, as well as cardiac and neurological disorders, similar to a mitochondrial disease phenotype [9].

Here, we evaluate the genetic, metabolic, and clinical characteristics of all reported patients with pathogenic variants in *UQCRC2* and describe two additional unrelated patients with recurrent episodes of hypoglycemia after flu-like illnesses, harboring the same missense variant in *UQCRC2 in trans* with a microdeletion encompassing the *UQCRC2* gene.

## 2. Materials and Methods

### 2.1. Literature Review

A literature review was performed to evaluate all clinical reports on patients diagnosed with pathogenic variants in *UQCRC2*. Demographic and clinical data were collected. Metabolic and mitochondrial function (including OXPHOS measurements) was evaluated.

Additional patients were recruited to the rare and undiagnosed research repository (IRB 19-005839) and genomic profiling of mitochondrial disease studies (IRB 19-003389). Deidentified fibroblast samples were analyzed for biomarker discovery, including functional studies by Seahorse respirometry and ETC complex enzymology measurements (IRB 16-004682).

### 2.2. Fibroblast Maintenance

Fibroblasts were maintained in Dulbecco’s Modified Eagle Media (DMEM) supplemented with 2 mM L-glutamine, 1 mM sodium pyruvate, 25 mM (4.5 g/L) glucose (Thermo Fisher Scientific Inc., Waltham, MA, USA), and 50 ng/mL uridine (Millipore Sigma, Burlington, MA, USA). Cells were maintained at 37 °C and 5% CO_2._ All fibroblasts were assayed between passage number 6 and passage number 8.

### 2.3. Respirometry

The Agilent Seahorse XF Cell Mito Stress Test (Agilent Technologies, Inc., Santa Clara, CA, USA) was used to investigate the oxygen consumption rate (OCR), extracellular acidification rate (ECAR), and proton efflux rate (PER) of cultured fibroblast cells, using ~10,000 cells per well seeded for 48 h, using a previously reported method [10]. OCR, ECAR, and PER were normalized to both cell number and citrate synthase (CS) activity. Before measuring respiration, the culture medium was replaced with XF DMEM (Agilent Technologies, Inc., Santa Clara, USA) supplemented with 10 mM XF glucose, 1 mM XF pyruvate, and 2 mM XF L-glutamine (Agilent Technologies, Inc., Santa Clara, CA, USA).

A detailed description of the methods is provided in Appendix A.1.

### 2.4. Mitochondrial Enzymology

The activities of mitochondrial complexes I (CI), II (CII), III (CIII), and IV (CIV) were assayed using a spectrophotometric enzyme activity assay [11] performed on a FLUOstar Omega spectrophotometric plate reader (BMG Labtech Inc., Cary, NC, USA). In total, ~5–10 million cultured skin fibroblasts were homogenized in 20 mM Tris-HCl (pH 7.6) (Thermo Fisher Scientific Inc., Waltham, MA, USA), using a bead mill homogenizer (Thermo Fisher Scientific Inc., Waltham, MA, USA) and 1.5 mL microtubes pre-filled with 1.4 mm ceramic beads (Omni International, Inc., Kennesaw, GA, USA). ETC complex activities for patient 1 were measured and normalized to CS activity and protein concentration using previously reported methods [12]. A detailed description of the methods is provided in Appendix A.2. ETC activity for patient 2 was performed by Baylor College of Medicine laboratories, with results normalized to protein concentration.

### 2.5. Patients

#### 2.5.1. Patient 1

**Figure 1:**
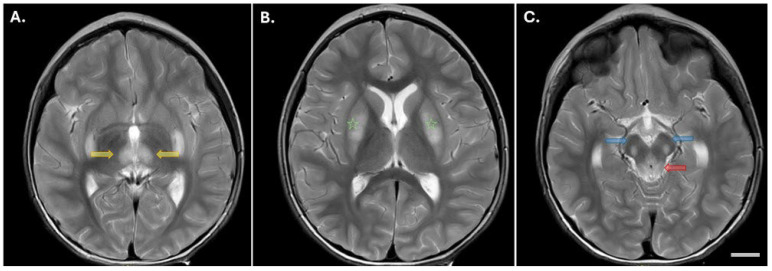
MRI findings in patient 1. (**A**–**C**) Brain MRI T2 axial section showing abnormal symmetric signals within the medial portion of the thalamus (yellow arrows); (**B**) within both the caudate and putamen (green star), and (**C**) within the substantia nigra (blue arrows) and periaqueductal gray matter (red arrow). Scale bar = 15 mm.

This male individual was born to healthy, non-consanguineous parents, was delivered vaginally without complications, and exhibited normal growth parameters at birth. The patient displayed episodes of vomiting, reduced activity, decreased consciousness, and tachypnea at 25 h after birth. Additional findings included hypoglycemia (lowest recording: 46 mg/dL; normal: >70 mg/dL), hyperammonemia (highest recording: 780 μmol/L; normal: <28 μmol/L), and lactic acidosis (highest recording: 4 mmol/L; normal: <2.1 mmol/L). Intermittent dicarboxylic aciduria and abnormalities in the acylcarnitine profile were also observed during further evaluation. Starting at 7 months of age, he began experiencing frequent episodes of metabolic decompensation triggered by infections. These episodes were characterized by vomiting, hypoactivity, lowered consciousness, and tachypnea, along with metabolic acidosis, jaundice, and elevated transaminases (AST and ALT >5–8 times above the upper limit of controls). At 6 years of age, the patient presented with mild-to-moderate developmental delay, hypotonia, and subtle dystonia. He has experienced 15 episodes of metabolic decompensation throughout his life, 5 of which occurred before his first birthday. Genome sequencing (GS) revealed the NM_003366.4(*UQCRC2*): c.[361T>C] p.[(Tyr121His)] missense variant *in trans* with a 16p12.2 deletion encompassing the entire *UQCRC2* gene (Seq[GRCh38] del(16)(p12.2p12.2)NC_000016.10:g(21936825_22429665)del). Magnetic resonance imaging (MRI) findings showed bilateral lesions with symmetrical distribution in multiple brain regions, including the medial portion of the thalamus (Figure 1A), hypothalamic region, putamen, caudate nucleus (Figure 1B), substantia nigra, periaqueductal gray matter (Figure 1C), pontine tegmentum, mesencephalic, and bulbar regions. Additionally, there was an observation of inferior vermis hypoplasia and a seemingly enlarged fourth ventricle. Coenzyme Q10 (CoQ10) was prescribed. He was diagnosed with abdominal migraine at age 4 years and put on amitriptyline.

#### 2.5.2. Patient 2

The second patient is a female born to healthy non-consanguineous parents via spontaneous vaginal delivery, with normal growth parameters. She had no notable health concerns during early childhood, and her growth and developmental milestones progressed normally. At 5 years of age, she experienced her first significant health episode, which presented with hypoglycemia (lowest recording: 42 mg/dL; normal: >70 mg/dL). This episode escalated to liver failure, characterized by synthetic liver dysfunction (evidenced by increased international normalized ratio (INR) and prothrombin time (PT)) and decreased factor V levels), hyperammonemia (highest recording: 233 μmol/L; normal: <28 μmol/L), and severe lactic acidosis (highest recording: 17.5 mmol/L; normal: <2 mmol/L). This condition was accompanied by encephalopathy necessitating an 18-day hospitalization. She experienced an additional significant life event at 8 years of age following a bout of Streptococcal pharyngitis. This episode was marked by altered mental status, hyperammonemia (225 μmol/L; normal: <28 μmol/L), recurrent hypoglycemia, and metabolic acidosis, which was managed with glucose infusion. The frequency of hypoglycemic episodes substantially decreased after adopting a regimen of frequent meals and fasting avoidance, with no new episodes occurring between ages 11 and 13.5 years. At 13 years of age, the patient demonstrates normal motor and speech development

**Figure 2:**
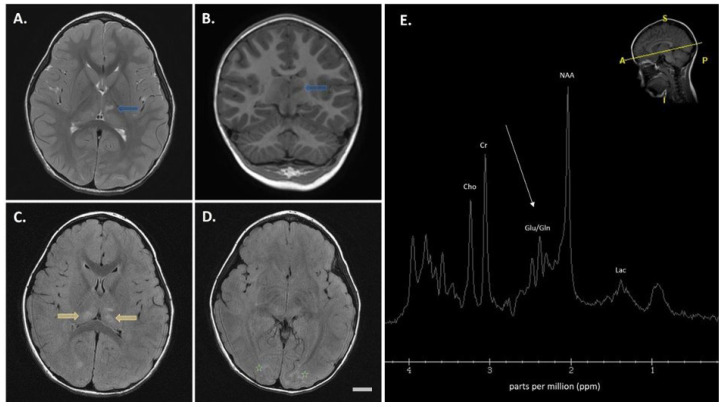
Brain magnetic resonance imaging (MRI) findings in patient 2. (**A**,**B**) Axial T2-weighted (**A**) and coronal T1-weighted (**B**) images show an abnormal signal in the anterior left thalamus (blue arrows). (**C**,**D**) Subtle signal alterations are also observed in the bilateral dorsal thalami (yellow arrows) (**C**) and bilateral occipital lobes (green asterisks) (**D**). (**E**) Magnetic resonance (MR) spectroscopy reveals a prominent glutamate/glutamine (Glu/Gln) peak at 2.2–2.4 parts per million (ppm) (white arrow), with a normal lactate (Lac) peak at 1.3 ppm. Inset shows a sagittal T1-weighted image with oblique anterior-posterior voxel acquisition (A = anterior, P = posterior, S = superior, I = inferior). Abbreviations: Lac, lactate; NAA, N-acetylaspartate; Glu/Gln, glutamate/glutamine; Cr, creatine; Cho, choline. Scale bar = 15 mm.

Exome sequencing (ES) identified the NM_003366.4(*UQCRC2*): c.[361T>C] p.[(Tyr121His)] missense variant *in trans* with a 16p12.2 deletion encompassing the entire *UQCRC2* gene (Seq[GRCh37] del(16)(p.12.2) NC_000016.10:g(21963507_22336556)del). Trio sequencing identified the heterozygous *UQCRC2* c.361T>C variant in the proband’s father, while no variants in the *UQCRC2* gene were identified in the proband’s mother. Both parents tested negative for the 16p12.2 deletion, suggesting a *de novo* deletion in the proband, though germline mosaicism is a possibility. An MRI obtained during the first decompensation episode revealed mild bilateral restricted diffusion in the thalamus and subtle signal alterations in the bilateral occipital lobes (Figure 2A–D). Magnetic resonance (MR) spectroscopy showed an elevated glutamate/glutamine peak with a normal lactate peak (Figure 2E), which may be indicative of hyperammonemic encephalopathy [13].

## 3. Results

### 3.1. Review of Previously Published Cases

The phenotypes of 14 individuals, including 12 previously reported and 2 unreported cases, were examined. This group comprised 6/14 (42%) males, 7/14 (50%) females, and 1/14 (7%) individuals whose gender was not reported. The onset of symptoms occurred neonatally in 6/14 (42%) patients, and between the ages of 1.5 and 4 years in another 6/14 (42%), with 2/14 (14%) patients lacking a detailed description of symptom onset. Diagnosis was made between the ages of 3.5 and 22.5 years in the reported cases, while 5/14 (36%) patients lacked specific diagnostic age details. Notably, consanguinity was present in 7/14 (50%) patients. Additionally, 1/14 (7%) had possessed a homozygous missense variant without reported consanguinity. All 14 (100%) patients experienced metabolic decompensation and/or encephalopathy, characterized by hypoglycemia, lactic acidosis, hyperammonemia, and liver failure (Table 1). A total of 2/14 (14%) patients were reported to have severe encephalopathy with Leigh-like syndrome. In total, 3/14 (21%) patients experienced generalized seizures during metabolic decompensation events, and myoclonic epilepsy was noted in 1/14 (7%). Delayed motor milestones and delayed speech were observed in 6/14 (42%) and 4/14 (29%), respectively. Mild to moderate intellectual disability was reported in 4/14 patients (29%). In total, 8/14 patients (57%) required interventions such as physical, occupational, or speech therapy. Additionally, microcephaly was identified in 1/14 (7%). This study shows an average follow-up period of 9.14 years (between 4 and 18.9 years), during which 11/14 (78%) patients displayed normalized liver function, 3/14 (21%) displayed residual neurological symptoms, 1/14 (7%) displayed severe hepatic failure, 1/14 (7%) displayed chronic developmental delay, and 1/14 (7%) died.

Neuroimaging results revealed diverse abnormalities in 9/14 (64%), including lateral ventriculomegaly, arachnoid and pineal cysts, inferior vermis hypoplasia, necrotic lesions in the basal ganglia and/or brain stem, and small parietal and temporal infarcts. Neurological symptoms varied, encompassing hyperreflexia, hyporeflexia, hemiparesis, cerebellar syndrome, and hypotonia. While hearing and vision were generally unaffected, 1/14 (7%) displayed mild sensorineural hearing loss, and 1/14 (7%) displayed bilateral vision impairment rated 6/10. Additionally, 1/14 (7%) exhibited divergent strabismus, 1/14 (7%) had atrial septal defect, and 1/14 (7%) experienced tuberculous meningitis, while 1/14 (7%) presented with flat feet. 1/14 (7%) displayed a small, enhanced focus on MRI at the liver’s dome, indicating a possible incidental hemangioma. Genetic testing revealed 8/14 (57%) homozygous variants and 6/14 (42%) compound heterozygous variants, with the most common variant being the homozygous NM_003366.4: c.[547C>T]; p.[(Arg183Trp)] variant present in 6/14 (42%).

OXPHOS assessments indicated a reduced CIII activity in 8/14 (57%), normal activity in 2/14 (14%), and no CIII activity data reported in 4/14 patients (29%). Respirometry results varied, with 1/14 (7%) patients displaying reduced OCR, 1/14 (7%) displaying normal OCR, 4/14 (29%) displaying substrate-dependent OCR variability, and 8/14 patients (57%) with no OCR data reported.

Metabolically, 8/14 patients (57%) displayed increased serum alanine levels, while 4/14 (29%) showed normal levels, and 2/14 (14%) reported no results. Serum tyrosine and proline levels were elevated in 3/14 (21%). Serum lactate levels were elevated at least in one episode in 13/14 (93%), with no reported results in one patient. Serum carnitine levels were largely unreported, though 2/14 (14%) displayed increased carnitine. Urinalysis indicated elevated urine ketone body levels in 7/14 (50%), normal in 2/14 (14%), and no reported testing for 5/14 (35%) patients. Table 1 comprehensively details the first decompensation event, developmental features, management, and outcomes for all 14 patients. Additional details, including demographic information, genetic test results, and labs and imaging findings, are presented in Table A1.

### 3.2. Patient Fibroblast Mitochondrial Enzymology

**Figure 3:**
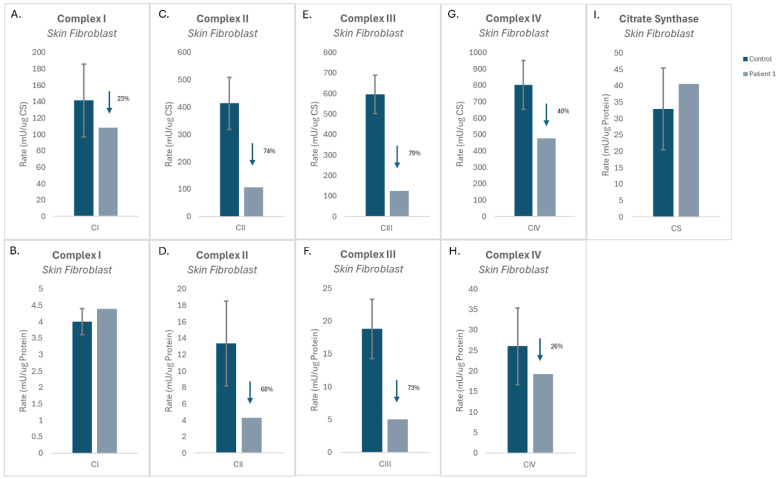
Mitochondrial enzymology in patient 1 cultured skin fibroblasts. (**A**–**H**) Activities of complexes I, II, III, and IV normalized to either citrate synthase (**A**,**C**,**E**,**G**) or protein concentration (**B**,**D**,**F**,**H**). (**I**) Citrate synthase activity relative to protein concentration. Data are represented as the mean ± standard deviation.

**Figure 4:**
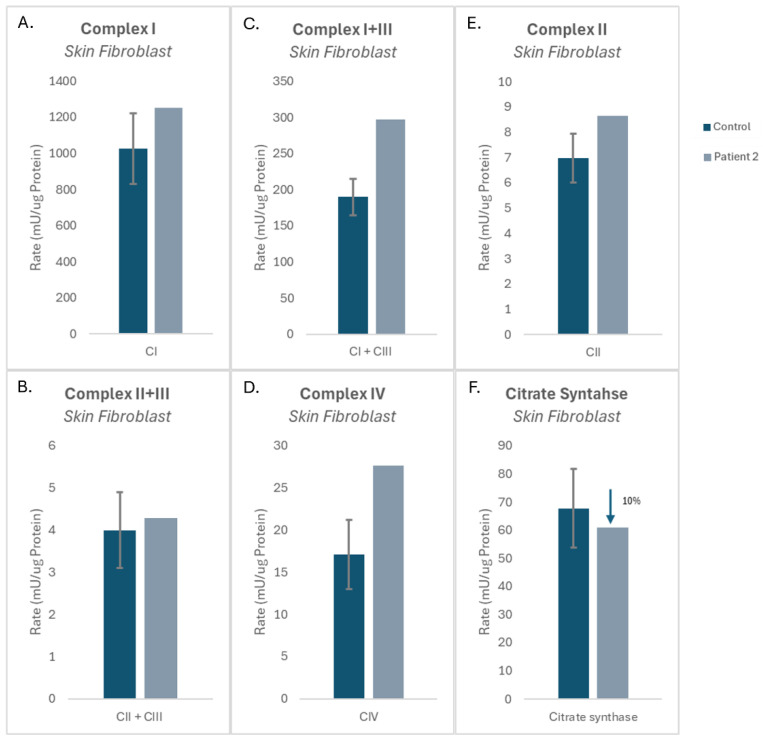
Mitochondrial enzymology in patient 2 cultured skin fibroblasts. (**A**–**E**) Activities of mitochondrial electron transport chain complexes I, I + III, II, II + III, and IV normalized to protein concentration. (**F**) Citrate synthase activity normalized to protein concentration. Data are represented as the mean ± standard deviation.

Mitochondrial ETC complex activities were assessed in cultured fibroblasts isolated from patient 1 and compared to three control fibroblast cell lines. No variation in CI activity was observed in patient 1 fibroblasts, when CI activity was normalized to both CS activity and protein concentration (Figure 3A,B). In contrast, patient 1’s fibroblasts showed notable reductions in CII activity when normalized to both CS activity and protein concentration (−3.75 SD and −2.03 SD of the mean, respectively) (Figure 3C–D). Patient 1 fibroblasts also displayed reduced activity of CIII (−5.8 SD and −3.52 SD of the mean) when normalized to CS activity and protein concentration, respectively (Figure 4E,F). Patient 1 fibroblasts displayed a reduction in CIV activity when normalized to CS activity (−2.55 SD of the mean; see Figure 3G), whereas CIV activity normalized to protein concentration remained unchanged (Figure 3H). CS activity relative to protein concentration, a proxy readout for mitochondrial mass, was almost normal, suggesting no change in the mitochondrial mass in patient 1 fibroblasts relative to controls (Figure 3I).

Patient 2 fibroblasts displayed normal CI activity relative to the controls (Figure 4A). CI + CIII showed 156% increase (+4 SD) relative to controls (Figure 4C). CII individual activity and CII combined with CIII (CII + CIII) activity showed comparable results to those of the controls (Figure 4B,E). CIV activity was elevated by 161% (+2.5 SD) relative to the control mean (Figure 4D). Citrate synthase activity levels were normal (Figure 4F).

### 3.3. Patient Fibroblast Respirometry

**Figure 5:**
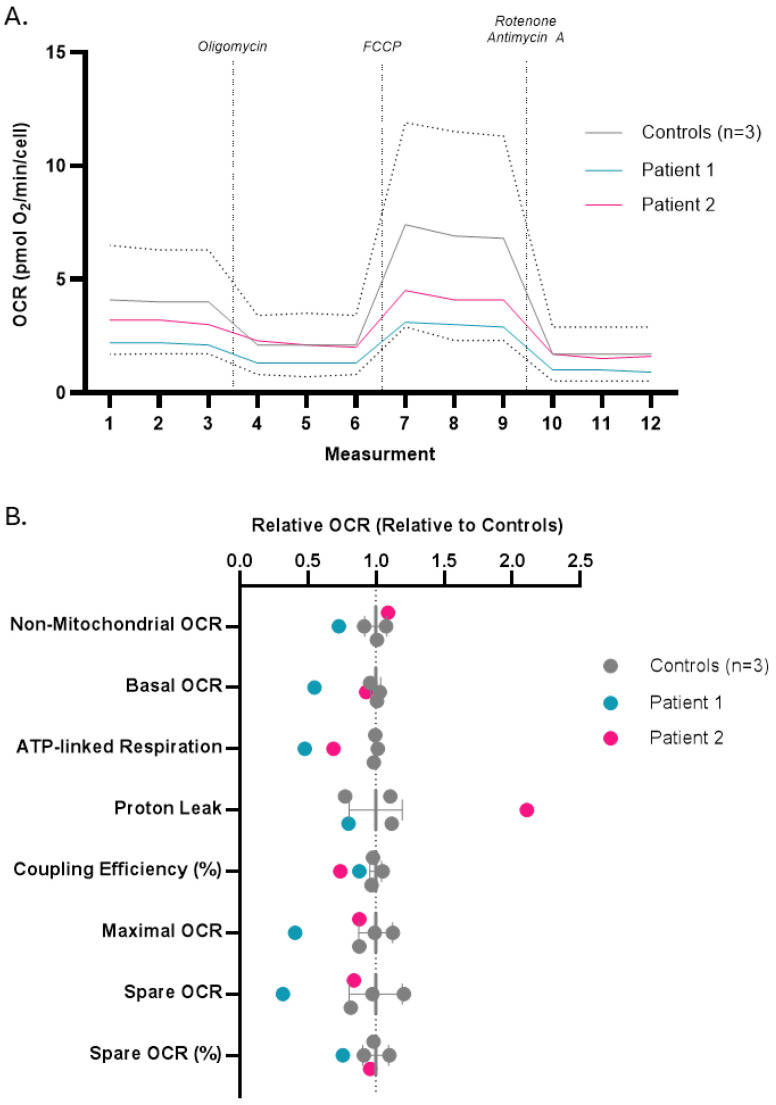
Seahorse respirometry in patients 1 and 2. (**A**) Oxygen consumption rates (OCRs) in patients and controls. The dotted lines represent the 95% confidence interval of the mean of the controls. (**B**) Plots of non-mitochondrial respiration, basal respiration, ATP-linked respiration, proton leak, coupling efficiency (%), maximal OCR, spare OCR, and spare OCR (%). Data are represented as the mean of measurements, and error bars represent 95% confidence interval of the mean of the controls. FCCP: carbonyl cyanide phenylhydrazone.

Seahorse respirometry was conducted on our *UQCRC2* patient fibroblasts as well as three healthy controls (Figure 5A). Patient 1 fibroblasts displayed a significant respiratory phenotype, characterized by profound reductions in non-mitochondrial OCR, basal OCR, ATP-associated OCR, coupling efficiency, maximal OCR, spare OCR, and spare OCR as a percentage of basal OCR relative to controls (Figure 5B). While patient 2 fibroblasts displayed a much milder phenotype; they also displayed a reduced ATP-associated OCR and coupling efficiency, with a concomitant increase in proton leak-associated OCR relative to the controls.

To better resolve the respiratory and enzymology phenotypes of our novel *UQCRC2* individuals, we incubated *UQCRC2* and 13 control fibroblasts in either 10 mM glucose or 10 mM galactose for 24 h and repeated Seahorse respirometry and mitochondrial enzymology. Galactose supplementation has previously been shown to drive mitochondrial metabolism, assisting in resolving metabolic dysfunction phenotypes [14].

**Figure 6:**
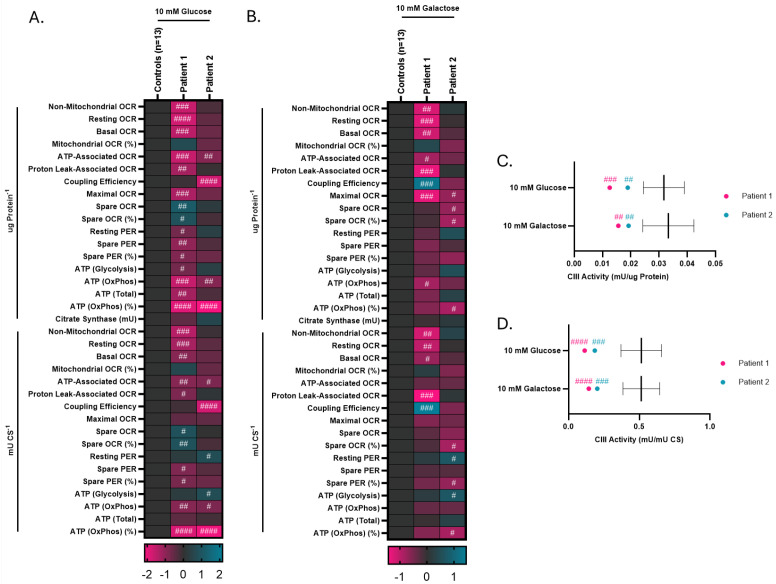
Seahorse respirometry and complex III (CIII) activity in glucose- and galactose-supplemented fibroblasts, normalized to both protein concentration and citrate synthase (CS) activity. (**A**,**B**) Heatmap of Seahorse respirometry readouts in control and *UQCRC2* fibroblasts supplemented with 10 mM glucose (**A**) or galactose (**B**) for 24 h. Standard deviations from the mean of the controls are plotted. (**C**,**D**) CIII activity in control and *UQCRC2* fibroblasts supplemented with either 10 mM glucose or 10 mM galactose, normalized to either protein concentration (**C**) or CS (**D**) activity. The mean and 95% confidence interval of the controls (n = 12) are plotted. #: >/<95% confidence interval of the mean of the controls (CIM); ##: >/<99% CIM; ###: >/<99.9% CIM; ####: >/<99.99% CIM.

Consistent with previous findings, patient 1 again showed a severely impaired respiratory phenotype. Patient 1 displayed reduced overall OCR when normalized to either protein concentration or CS activity when supplemented with 10 mM glucose (Supplementary Figure S1A) and displayed several respirometry readouts below the 95% confidence interval of the mean of the controls (CIM), including non-mitochondrial, resting, basal, ATP-associated, proton-leak associated, and maximal OCRs (Figure 6A). Both resting and spare PER were also reduced, consistent with reduced glycolytic flux (Figure 6A). Notably, while both glycolysis and OXPHOS-derived ATP production rates were reduced, the percentage of total ATP produced via OXPHOS was very highly depleted (Figure 6A).

While the overall OCR in patient 2 was well within the 95% CIM normalized to either protein concentration or CS activity when supplemented with 10 mM glucose (Supplementary Figure S1C), patient 2 nevertheless displayed multiple respiratory anomalies, including a large reduction in coupling efficiency (Figure 6A). Notably, as with patient 1, patient 2 displayed reduced ATP-associated OCR, reduced OXPHOS-derived ATP production, as well as a profound reduction in the OXPHOS-derived ATP production as a percentage of total ATP production (Figure 6A).

As expected, incubation with 10 mM galactose had profound effects on respiration in the control fibroblasts, reducing coupling efficiency as well as the percentage of ATP produced via OXPHOS, and strongly inducing PER, consistent with an increased glycolytic flux (Supplementary Figure S1E). Supplementation with 10 mM galactose had relatively little effect on the respiratory profile of patient 1, which continued to display reduced non-mitochondrial, resting, basal, ATP-associated, proton-leak associated and maximal OCR, as well as reduced OXPHOS ATP production (Figure 6B). Conversely, supplementation with 10 mM galactose revealed additional respiratory anomalies in patient 2 fibroblasts, including reduced maximal and spare OCR, consistent with a defect in the mitochondrial respiratory chain (Figure 6B).

Both patient 1 and patient 2 displayed reduced CIII activity relative to 12 control fibroblast lines when normalized to both protein concentration and CS activity, whether supplemented with 10 mM glucose or galactose (Figure 6C,D).

## 4. Discussion

*UQCRC2*, located at chromosome 16p12.2, encodes a core subunit of mitochondrial respiratory chain CIII, essential for electron transport and oxidative phosphorylation [1,2]. Biallelic pathogenic variants in *UQCRC2* have been associated with mitochondrial disease characterized by lactic acidosis, developmental delay, hepatopathy, and episodic metabolic decompensation [3,4,5,6].

In this study, we report two unrelated patients with biallelic *UQCRC2* variants, both carrying the same missense variant (c.361T>C, p.Tyr121His) *in trans* with a *UQCRC2*-encompassing 16p12.2 microdeletion. While the c.361T>C variant was initially classified as a variant of uncertain significance (VUS), it has since been reclassified as likely pathogenic by GeneDx (https://www.ncbi.nlm.nih.gov/clinvar/RCV000493198/ (accessed on 18 March 2026)), based on emerging evidence. In both of our patients, the variant is associated with decreased CIII activity, impaired cellular respiration and ATP production, and multiple biochemical features consistent with *UQCRC2*-related disease.

To date, 14 individuals with pathogenic or likely pathogenic *UQCRC2* variants have been reported, including our two patients. Among the 10 patients with available mitochondrial complex activity measurements, 8 showed decreased CIII activity [6,7], reinforcing the strong biochemical signature of *UQCRC2* loss-of-function. Notably, we were only able to resolve reduced CIII activity in patient 2 against a relatively large cohort of controls; however, Seahorse analysis consistently revealed respirometry associated with significantly reduced ATP production, pointing toward subtle or tissue-specific mitochondrial dysfunction. This highlights the limitations of fibroblast-based assays and the importance of complementary functional analyses in diagnosis.

Pathogenic *UQCRC2* variants are known to impair CIII dimerization, a key step in its early assembly [15]. In previous studies, this dysfunction has been reflected in elevated levels of caseinolytic mitochondrial matrix peptidase (CLPP) in patient-derived fibroblasts, a compensatory response to defective complex assembly. Elevated CLPP has been observed in other *UQCRC2*-deficient individuals, and although we did not assess it here, it remains a promising mechanistic biomarker of defective CIII biogenesis.

The clinical presentations of our patients align with the known spectrum of CIII deficiency, characterized by metabolic crises such as lactic acidosis and hyperammonemia. The most common form of CIII deficiency, BCS1L-related mitochondrial disease, caused by variants in the CIII chaperone protein BCS1L, also presents with metabolic crises, including lactic acidosis, aminoaciduria, and hypoglycemia, as well as neurologic symptoms, viz. seizures and movement disorders, and hepatopathy [16,17], very similar to the phenotype observed in our *UQCRC2* cohort. In our extended cohort of 14 patients, 13 had elevated serum lactate (average 10.65 mmol/L), 11 had hyperammonemia (average 300.1 µmol/L), and 8 had increased serum alanine, findings strongly suggestive of mitochondrial dysfunction. Importantly, high lactate alone is not specific for mitochondrial disease, but levels above 10 mmol/L, especially in the absence of cardiac disease or sepsis, raise suspicion. Hyperammonemia, while rare in mitochondrial disorders, appears more common in *UQCRC2* deficiency and may reflect a metabolic pattern similar to TMEM70-related disorders [18].

Neuroimaging in both patients showed altered diffusion in subcortical structures (thalamus and basal ganglia) and lactate peaks, indicative of cytotoxic or interstitial edema. These findings support the presence of energy failure during decompensation and align with patterns seen in other mitochondrial encephalopathies [19].

Assessment of mitochondrial respiration by Seahorse analysis has been performed in only 8 of 14 reported cases. Among these, results were variable: decreased oxygen consumption in 1/8 (12.5%) [5], normal in 1/8 (12.5%) [4], substrate-dependent variation in 4/8 (50%) [4], and reduced ATP-linked respiration in 2/8 (25%). In our study, both patients demonstrated evidence of significantly reduced mitochondrial ATP production. Notably, patient 1, who displayed a significantly more severe clinical phenotype, also displayed a much more profound biochemical and respiratory phenotype. Interestingly, despite a less severe reduction in CIII activity and a less profound respiratory phenotype, patient 2 also displayed a severe reduction in mitochondrial ATP production and did indeed display a reduced maximal respiration upon galactose stress, consistent with a defect in the mitochondrial respiratory chain.

An important novel observation in our study is the co-occurrence of a 16p12.2 microdeletion in both patients. This region includes *UQCRC2* as well as several other genes (e.g., *PDZD9*, *MOSMO*, *EEF2K*, and *POLR3E*), none of which have been linked to the observed phenotype. Meanwhile, larger deletions (~520 kb) encompassing this locus are associated with the 16p12.2 microdeletion syndrome, characterized by variable developmental delay, seizures, and congenital anomalies [9]. These are often inherited from asymptomatic parents and show incomplete penetrance, likely influenced by underlying segmental duplications (BP1–BP3) that predispose the region to structural rearrangements [20]. This study represents the first reported association between the 16p12.2 microdeletion and acute metabolic decompensations. We do not propose that the microdeletion alone is responsible for the phenotype. Rather, we propose that the deletion of *UQCRC2 in trans* with a missense variant in the same gene explains the observed clinical presentation of our patients. Although both patients harbor deletions at this locus and experienced recurrent metabolic crises, the limited sample size and variability in gene content preclude definitive conclusions about causality. Based on available evidence, we hypothesize that the clinical phenotype is primarily driven by biallelic disruption of *UQCRC2*, with the microdeletion contributing through loss of *UQCRC2* rather than broader regional effects. Nevertheless, the presence of the 16p12.2 deletion in patients with unexplained lactic acidosis or metabolic decompensations should prompt targeted sequencing of *UQCRC2*.

We acknowledge that our study does not include mechanistic modeling of the missense variant or deletion, such as CIII assembly assays or rescue experiments. However, this work aimed to consolidate reported clinical and biochemical features and assess functional consequences using diagnostic assays. Future studies employing genetic models and targeted assays will be essential to fully elucidate the pathogenic mechanisms of *UQCRC2* dysfunction.

Therapeutically, metabolic decompensations were frequently triggered by febrile illness, and glucose infusion was effective in stabilizing patients, even in the absence of hypoglycemia [4,5,6]. Preventive strategies, including frequent meals, avoidance of fasting, and carbohydrate-rich diets, were implemented in several cases [4,21,22]. Pharmacologic treatment with ubidecarenone (CoQ10) has been trialed in at least seven patients, with some reporting decreased hospitalizations, though its effect remains difficult to isolate from age-related improvement [4].

In conclusion, we expand the phenotypic spectrum (including vermis hypoplasia and basal ganglia involvement) and genetic spectrum of *UQCRC2* deficiency by describing two additional patients with compound heterozygous variants involving the 16p12.2 microdeletion and a missense variant. Our findings underscore the consistent biochemical hallmarks of the disorder, the diagnostic relevance of respiratory chain and Seahorse assays, and the potential role of the 16p12.2 deletion as a diagnostic signal. Further mechanistic and segregation studies are needed to clarify the contribution of the deletion, refine genotype–phenotype correlations, and improve therapeutic strategies.

Although internal organ involvement beyond the liver has not been reported in *UQCRC2* deficiency, comprehensive organ screenings are recommended at diagnosis due to the small number of cases [23]. Key evaluations should include neurological assessments via MRI and electroencephalogram (EEG), as well as liver function tests. Common lab abnormalities during acute metabolic decompensation include hypoglycemia, lactic acidosis, hyperammonemia, elevated pyruvate, low bicarbonate, and increased blood alanine. OCR testing in our patients showed reduced ATP-linked respiration, suggesting impaired energy metabolism. Given limited OCR data, expanding this testing to fibroblasts in all newly diagnosed patients is advised. Preventive strategies, such as fasting avoidance and glucose infusions, have proven effective in preventing acute decompensation. For mild symptoms like developmental delay or muscle tone changes, treatment should include physical, occupational, and speech therapies, alongside dietary management by a metabolic dietitian. While high-dose CoQ10 is considered safe, further research is needed to confirm its therapeutic benefits. A multidisciplinary approach combining medical, nutritional, and therapeutic care is essential for managing *UQCRC2* deficiency.

## 5. Conclusions

*UQCRC2* deficiency is marked by recurrent liver failure, lactic acidosis, hypoglycemia, hyperammonemia, and sometimes encephalopathy. The severity and frequency of metabolic decompensations largely determine outcomes. Neurological symptoms like tremors, cerebellar syndrome, hypotonia, and reflex variations are common, but unlike other mitochondrial disorders, *UQCRC2* deficiency typically lacks neurodegenerative progression and responds well to glucose infusion, suggesting a potentially less severe prognosis than other CIII deficiencies. Clinicians should consider *UQCRC2* deficiency in differential diagnoses of similar presentations, with genetic testing essential to confirm the diagnosis and exclude other genetic causes. Strengthening collaborations between families, researchers, and clinicians could improve understanding of the disorder and lead to better diagnostic and treatment strategies.

## Figures and Tables

**Table 1:** Clinical features and management plans of individuals with UQCRC2 variants reported in the literature.

	Paper	Bansept et al., 2022 [4]						Gaingarrd et al., 2017 [5]	Burska et al., 2021 [7]	Ogawa et al., 2020 [8]	Miyake et al., 2012 [6]			Novel Cases	
Initial presentation and metabolic changes	Onset age	15 m	5 d	15 m	19 m	4.1 y	3.5 y	1 d	N/A	N/A	1 d	1 d	18 m	25 h	5 y
	Symptoms and laboratory changes	Gastro-enteritis	Neonatal period	Vomiting	Treatment with sodium valproate	Tonsillectomy	Fever, food refusal	Neonatal period	N/A	N/A	Intercurrent illness	Upper RTI	N/A	Presumed sepsis	Flu-like illness
	Lactic acidosis	+	+	+	+	+	+	+	-	-	+	+	- ^#^	+	+
	Hypo-glycemia	+	+	+	+	+	+	+	+	-	+	+	+	+	+
	Hyper-ammonemia	+	+	+	+	+	+	+	-	-	+	+	-	-	+
	Ketosis	+ in 4 patients/N/A in 3							N/A	N/A	+	+	+	-	-
	Elevated serum alanine	+ in 3 patients							N/A	N/A	+	+	+	+	+
	Liver failure	+	+	+	+	+	+	+	-	-	-	-	-	+	+
	Neurological symptoms	+	+	+	+	+	+	+	+ ^1^	+ ^1^	-	+	+	+ ^1^	+ ^1^
	Interval to genetic diagnosis (y)	10	7	2	13	7	19	10	N/A	N/A	5	N/A	N/A	6	5
	CIII activity	↓	↓	↓	↓	↓	↓	↓	N	N/A	↓	N/A	N/A	↓	N
Development	Motor delay	4 patients/7 patients							+	+	N	+	+	+	+
	IQ	N	N/A	N/A	↓	↓↓	N/A	↓	↓↓	N/A	↓↓	↓↓	N/A	N	N
	Neurological symptoms	Tremor, cerebellar syndrome, dyskinesia	Left arm hemiparesis, hypotonia	N	Hyperreflexia, neuropathic pain in limbs, seizure	Hyperreflexia, dysmetria, seizure	N	Hyporeflexia and fatigability, hypotonia	Divergent strabismus, intention tremor, unsteady walking	Leigh-like syndrome	N	Seizure	Seizure	Hypotonia and subtle dystonia	N
Management and prognosis	Intravenous glucose	+ ~	+ ~	+ ~	+ ~	+ ~	+ ~	+ ~	N/A	N/A	+ ^2^	+ ^2^	+ ^2^	+ ~^3^	+ ^3^
	Hospitalizations frequency	>20	5	>15	>25	15	>30	>50	N/A ^	N/A ^	>10	>10	N/A	15	2
	Follow-up years	10.2	1.1 *	4.4	13	7.8	18.9	14.1	6.4	N/A	5	4	N/A	6	8
	Liver failure	-	+ ^	-	-	-	-	+	-	N/A	-	-	-	-	-
	Neurological sequelae	+	N/A	-	+	+	-	-	+ ≈	N/A	-	-	-	-	-

m: months, y: years, d: days, h: hours, RTI: respiratory tract infection, IQ: intelligence quotient, N/A: not applicable, N: normal. ((+) = present, (-) = absent symptoms), (↓ = mild, ↓↓ = severe decrease/delay.) ^#^ Hyperlactemia. ^ Leigh syndrome. * Died. ~ Trial of coenzyme Q10. ≈ Continuous developmental delay. ^1^ Encephalopathy. ^2^ In total, 60% of calories from carbohydrate, 30% of calories from fat. ^3^ At a dose of 2.8 g/kg/day, divided into 3 doses.

## Data Availability

The original contributions presented in this study are included in the article/Supplementary Materials. Further inquiries can be directed to the corresponding author.

## Supplementary Materials

The following supporting information can be downloaded at: https://www.mdpi.com/article/10.3390/cells15070596/s1, Figure S1. Seahorse respirometry in glucose- and galactose-supplemented UQCRC2 and control (n = 13) fibroblasts, normalized to both protein concentration and citrate synthase (CS) abundance.

## Abbreviations

The following abbreviations are used in this manuscript:

*UQCRC2*	Ubiquinol–cytochrome C reductase core protein 2
MRI	Magnetic resonance imaging
MR	Magnetic resonance
OCR	Oxygen consumption rate
ECAR	Extracellular acidification rate
PER	Proton efflux rate
OXPHOS	Oxidative phosphorylation
CS	Citrate synthase
ETC	Electron transport chain
VUS	Variant of uncertain significance
ES	Exome sequencing
GS	Genome sequencing
INR	International normalized ratio
PT	Prothrombin time
CLPP	Caseinolytic mitochondrial matrix peptidase proteolytic subunit
ATP	Adenosine triphosphate
CI	Complex I
CII	Complex II
CIII	Complex III
CIV	Complex IV
AST	Aspartate transferase
ALT	Alanine transaminase
CoQ10	Coenzyme Q10
CIM	Confidence interval of the mean of the controls
EEG	Electroencephalogram
FCCP	Carbonyl cyanide phenylhydrazone
DTNB	5,5’-Dithiobis-(2-nitrobenzoate)
DMEM	Dulbecco’s modified Eagle medium
NADH	Nicotinamide adenine dinucleotide
BSA	Bovine serum albumin
DCPIP	2,6-Dichlorophenolindophenol
DUB	Decylubiquinone
EDTA	Ethylenediaminetetraacetic
NaAz	Sodium azide
Cytochrome C	CytC

## Appendix A

### Appendix A.1. Methods for OCR Measurements

The Agilent Seahorse XF Cell Mito Stress Test was used to investigate the oxygen consumption rate (OCR) of fibroblast cell lines. The OCR was assessed with the Seahorse XFe96 Extracellular Flux Analyzer (Agilent, Santa Clara, CA, USA) using 10,000 cells per well seeded for 48 h, as previously described [10]. Before measuring respiration, the culture medium was replaced with XF DMEM (Agilent, Santa Clara, CA, USA) supplemented with 10 mM glucose, 1 mM pyruvate, and 2 mM L-glutamine. Cells were assayed with the Seahorse XF Cell Mito Stress Test (Agilent, Santa Clara, CA, USA). Briefly, following three initial oxygen consumption rate (OCR) and extracellular acidification rate (ECAR) measurements, 2.5 μM oligomycin, 2.0 μM carbonyl cyanide phenylhydrazone (FCCP), and 0.5 μM rotenone and antimycin A were added sequentially, with three OCR and ECAR readings following each administration. FCCP concentrations were previously titrated to determine an optimal concentration. Each sample had eight experimental duplicates. Following the last respirometry measurement, wells were incubated with membrane-permeable nuclear staining compound Hoechst 33342 (Millipore Sigma, Burlington, MA, USA), and cells were counted using the BioTek Cytation 5 plate reader (Agilent, Santa Clara, CA, USA). Subsequently, cells were lysed by incubation with 20 μM Tris-HCl (Thermo Fisher Scientific Inc., Waltham, MA, USA) plus 10% Triton X-100 (Millipore Sigma, Burlington, MA, USA), and citrate synthase (CS) activity was measured. CS converts oxaloacetate and acetyl-CoA to citrate and S-CoA. The S-CoA produced by the CS reaction cleaves DTNB (colorless) to TNB^2-^ (yellow).

Lysates were incubated with acetyl-CoA (Millipore Sigma, Burlington, MA, USA), oxaloacetate, and 5,5’-dithiobis-(2-nitrobenzoate) (DTNB) (Millipore Sigma, Burlington, MA, USA), and the increasing absorbance at 412 nm was measured seven times over 14 min.

For the Seahorse assay of galactose-supplemented cells, fibroblasts derived from our two novel UQCRC2 individuals, as well as 13 healthy controls, were seeded in sextuplicate at 10,000 cells per well, and incubated for 48 h at 37 °C, 5% CO_2_ in DMEM supplemented with 2 mM L-glutamine, 1 mM sodium pyruvate, and 25 mM (4.5 g/L) glucose (Thermo Fisher Scientific Inc., Waltham, MA, USA). Subsequently, three wells of each cell line were replaced with DMEM supplemented with 2 mM L-glutamine, 1 mM pyruvate, (Thermo Fisher Scientific Inc., Waltham, MA, USA) and 10 mM D-(+)-glucose (Millipore Sigma, Burlington, MA, USA), while the media in the remaining 3 wells was replaced with media supplemented with 2 mM L-glutamine, 1 mM sodium pyruvate, and 10 mM D-(+)-galactose (Millipore Sigma, Burlington, MA, USA). 24 h later, the Seahorse XF Cell Mito Stress Test assay was performed as described above, with the exception that galactose-supplemented wells were run in XF DMEM supplemented with 10 mM galactose. ECAR readings were converted to PER using the Agilent Wave software (version 2.6.3.5) (Agilent, Santa Clara, CA, USA). Cells were solubilized in 10 mM Tris-HCl (Thermo Fisher Scientific Inc., Waltham, MA, USA) plus 10% Triton X-100 (Millipore Sigma, Burlington, MA, USA), and CS activity and protein concentration were assayed as proxy readouts for mitochondrial and cell mass, respectively.

The following respiratory readouts were calculated:

- Non-mitochondrial OCR: Oxygen consumption rate (pmol O_2_/min) from non-mitochondrial sources. Equal to the minimum OCR following rotenone and antimycin A administration.
- Resting OCR: Total oxygen consumption at resting conditions. Equal to the total OCR at the final time point before oligomycin administration.
- Basal OCR: Mitochondrial oxygen consumption at resting conditions. Equal to resting OCR minus the non-mitochondrial OCR.
- Mitochondrial OCR (%): Resting mitochondrial oxygen consumption as a percentage of the total resting oxygen consumption. Equal to the basal OCR divided by the resting OCR.
- ATP-associated OCR: The oxygen consumption associated with ATP synthase. Equal to the OCR at the final time point before oligomycin administration, minus the minimum OCR following oligomycin administration.
- Proton leak-associated OCR: Mitochondrial oxygen consumption following oligomycin administration. Equal to the minimum OCR following oligomycin administration minus the non-mitochondrial OCR.
- Coupling efficiency: The percentage of basal mitochondrial OCR that goes toward ATP production. Equal to the ATP-associated OCR divided by the basal OCR.
- Maximal OCR: The maximum OCR of the uncoupled mitochondrial respiratory chain (MRC). Equal to the maximum OCR following FCCP administration minus the non-mitochondrial respiration.
- Spare OCR: The difference between the maximal oxygen consumption rate of the MRC and the resting mitochondrial OCR. Equal to the maximal OCR minus the basal OCR.
- Spare OCR (%): Spare OCR as a percentage of basal OCR. Equal to the spare OCR divided by the basal OCR.
- Resting PER: The proton efflux rate (pmol H^+^/min) under resting conditions. Equal to the PER at the last measure before oligomycin administration.
- Spare PER: The difference between the resting and maximal glycolytic flux. Equal to the maximal PER following oligomycin administration and PER at the last measurement before oligomycin administration.
- Spare PER (%): Spare PER as a percentage of basal PER. Equal to spare PER divided by basal PER.
- ATP (Glycolysis): The ATP production rate (pmol ATP/min) from glycolysis. Equal to the resting PER.
- ATP (OXPHOS): The ATP production rate from mitochondrial oxidative phosphorylation. Equal to the ATP-associated OCR multiplied by five.
- ATP (Total): The total ATP production rate. Equal to ATP (Glycolysis) plus ATP (OXPHOS).
- ATP (OXPHOS) (%): The ATP production rate from mitochondrial oxidative phosphorylation as a percentage of the total ATP production rate, equal to ATP (OXPHOS) divided by ATP (Total).

All respirometry readouts were normalized to both protein concentration and citrate synthase activity to assess for respiratory changes relative to cell mass and mitochondrial mass, respectively.

### Appendix A.2. Methods for Mitochondrial Electron Transport Chain Complex Enzymology in Fibroblasts

Complex I (CI): Cell homogenates were incubated in a potassium phosphate (K_2_HPO_4_)-buffered solution (Millipore Sigma, Burlington, MA, USA) of bovine serum albumin (BSA) (Millipore Sigma, Burlington, MA, USA) in the presence of nicotinamide adenine dinucleotide (NADH) (Roche, Basel, Switzerland), ubiquinone (CoQ) (Millipore Sigma, Burlington, MA, USA), and the artificial electron receptor 2,6-dichlorophenolindophenol (DCPIP) (Millipore Sigma, Burlington, MA, USA). Ubiquinone reduced to ubiquinol (QH_2_) by the oxidation of NADH to NAD^+^ by CI rapidly reduces DCPIP (blue) to DCIPIH_2_ (colorless). The NADH oxidation was assayed through spectrophotometric measurement of the extinction of DCPIP absorption at 600 nm over 17 min. Non-specific NADH oxidation was determined by simultaneously assaying NADH oxidation in each tissue homogenate in the presence of the potent CI inhibitor rotenone (Millipore Sigma, Burlington, MA, USA), and CI activity was calculated by subtracting the non-specific (rotenone-inhibited) NADH oxidation from the total (rotenone-uninhibited) NADH oxidation.

Complex II (CII): Cell homogenates were incubated in a K_2_HPO_4_-buffered solution of BSA, ethylenediaminetetraacetic (EDTA) (Millipore Sigma, Burlington, MA, USA), and sodium azide (NaAz) (Millipore Sigma, Burlington, MA, USA) in the presence of succinate (Millipore Sigma, Burlington, MA, USA), decylubiquinone (DUB) (Millipore Sigma, Burlington, MA, USA), adenosine triphosphate (ATP) (Millipore Sigma, Burlington, MA, USA), and DCPIP. DUB, reduced to DUH_2_ by the oxidation of succinate by CII, rapidly oxidizes DCPIP (blue) to DCIPIH_2_ (colorless). Succinate oxidation was assayed through spectrophotometric measurement of the extinction of DCPIP absorbance at 600 nm for 15 min. Non-specific reduction of DCPIP was corrected for by simultaneously assaying each tissue homogenate in the presence of the potent CII inhibitor malonate (Millipore Sigma, Burlington, MA, USA), and CII activity was calculated by subtracting the non-specific (malonate-inhibited) succinate oxidation from the total (malonate-uninhibited) succinate oxidation.

Complex III (CIII): Cell homogenates were incubated in a K_2_HPO_4_-buffered solution of EDTA, NaAz, and polysorbate 20 (Millipore Sigma, Burlington, MA, USA) in the presence of reduced DUH2 and cytochrome C (CytC) (Millipore Sigma, Burlington, MA, USA). Reduction of CytC by CIII via oxidation of DUH_2_ was assayed via spectrophotometric measurement of reduced CytC absorbance at 550 nm over 15 min. Non-specific reduction of CytC was corrected for by first assaying for CytC reduction in the presence of DUH2, but in the absence of the homogenate.

Complex IV (CIV): Cell homogenates were incubated in a K_2_HPO_4_-buffered solution in the presence of reduced CytC. The oxidation of reduced CytC by CIV was assayed via spectrophotometric measurement of the extinction of absorption by reduced CytC at 550 nm over 15 min. The reaction endpoint was assessed by artificially oxidizing all reduced CytC in the reaction mixture via the addition of the potent oxidizer potassium ferricyanide (K3Fe(CN)6) (Millipore Sigma, Burlington, MA, USA), and the total reduced CytC in the reaction was quantified using a triplicate of blank wells lacking tissue homogenate.

Citrate synthase (CS): Cell homogenates were incubated in a Tris-HCl-buffered solution in the presence of oxaloacetic acid (Millipore Sigma, Burlington, MA, USA), acetyl-CoA (Millipore Sigma, Burlington, MA, USA), and DTNB (Millipore Sigma, Burlington, MA, USA). CoA-SH generated by the condensation of acetyl-CoA and oxaloacetic acid by CS to citric acid rapidly cleaves DTNB (colorless) to TNB^-^, which rapidly ionizes to TNB2^-^ (yellow). Condensation of acetyl-CoA and oxaloacetic acid by CS was assayed via spectrophotometric measurement of TNB2^-^ at 411nm over 15 min.

Protein concentration: Protein concentration of cell homogenates was assayed using the Pierce BCA Protein Assay (Millipore Sigma, Burlington, MA, USA).

For galactose-supplemented enzymology, fibroblasts derived from our two novel UQCRC2 individuals, as well as 12 healthy controls, were incubated for 24 h at 37C, with 5% CO_2_ in DMEM supplemented with 10 mM glutamine and 1 mM pyruvate, as well as either 10 mM glucose or galactose. Cells were collected and assayed for CIII activity as described above.

**Table A1:** Demographics, genetic findings, labs, and imaging in individuals with *UQCRC2* variants reported in the literature.

	**Paper**	Bansept et al., 2022 [4]						Gaingarrd et al., 2017 [5]	Burska et al., 2021 [7]	Ogawa et al., 2020 [8]	Miyake et al., 2012 [6]			Novel Cases	
Demographics and history	Patient number	1	2 *	3	4	5	6	7	8	9	10	11	12	13 (P1)	14 (P2)
	Gender	M	F	F	M	M	F	M	F	N/A	F	M	F	M	F
	Parent consanguinity	-	+	+	-	-	+	+	-	N/A	+	+	+	-	-
	Antenatal/neonatal history	IUGR/feeding difficulties	IUGR/decompensation	Hyperechogenic gut/normal	Gestational hypertension/mild jaundice	Gestational hypertension/transient hypoglycemia	IUGR/transient respiratory distress	IUGR/decompensation	Uneventful/uneventful	N/A	Pathological cardiotocogram	Uneventful/uneventful	Small for GA/mild RD	Uneventful/uneventful	Uneventful/uneventful
Genetic analysis findings	Coding sequence variant	c.[1330T>A]; [1087C>T]	c.[547C>T]; [547C>T]	c.[547C>T]; [547C>T]	c.[379C>T]; [whole gene deletion]	c.[379C>T]; [whole gene deletion]	c.[266 T>C]; [266 T>C]	c.[547C>T]; [547C>T]	c.[665G>C]; [665G>C]	c.[1340C>A]; [613-3->A]	c.[547C>T]; [547C>T]	c.[547C>T]; [547C>T]	c.[547C>T]; [547C>T]	c.[361T>C]; [whole gene deletion]	c.[361T>C]; [whole gene deletion]
	Protein change	p.[(Leu444Met)]; [(Gln363*)]	p.[(Arg183Trp)]; [(Arg183Trp)]	p.[(Arg183Trp)]; [(Arg183Trp)]	p.[(Arg127Trp)]; [(whole deletion)]	p.[(Arg127Trp)]; [(whole deletion)]	p.[(Leu89Pro)]; [(Leu89Pro)]	p.[(Arg183Trp)]; [(Arg183Trp)]	p.[(Gly222Ala)]; [(Gly222Ala)]	p.[(Thr447Lys)]	p.[(Arg183Trp)]; [(Arg183Trp)]	p.[(Arg183Trp)]; [(Arg183Trp)]	p.[(Arg183Trp)]; [(Arg183Trp)]	p.[(Tyr121His)]; [del (16)(p.12.2)]	p.[(Tyr121His)]; [del (16)(p.12.2)]
	ACMG classification (ClinVar)	Likely pathogenic	Likely pathogenic	Likely pathogenic	Likely pathogenic	Likely pathogenic	Likely pathogenic	Likely pathogenic	Pathogenic	N/A	Pathogenic	Pathogenic	Pathogenic	Likely pathogenic	Likely pathogenic
	Method of detection	NP	NP followed by familial analysis	ES	NP	NP followed by familial analysis	NP	NP	Trio-ES+ mitochondrial DNA analysis	N/A	ES with linkage analysis	ES with linkage analysis	ES with linkage analysis	GS	Trio-ES
Imaging	MRI signal abnormalities	+ ^ (BG)	+ ^ (BG)	N/A	-	-	-	-	+ ^ (BS)	N/A	+ (RP and RT)	-	-	+ (BS and BG)	+ (BG)
	Associated conditions	Myopia, strabismus, mild SNHL, micropenis	N/A	None	Arachnoid cyst operated on twice	TB meningitis, hypothyroidism, operated cholesteatoma	Asthma, pineal cyst.	Bilateral vision loss	Flat feet	N/A	Atrial septal defect and renal tubular acidosis	Adrenal insufficiency	None	Inferior vermis hypoplasia	Liver hemangioma

M: male, F: female, N/A: not applicable, IUGR: intrauterine growth restriction, GA: gestational age, RD: respiratory distress, NP: nuclear panel, ES: exome sequencing, GS: genome sequencing, SNHL: sensorineural hearing loss, TB: tuberculosis, BS: brain stem, BG: basal ganglia, RP: right parietal, RT: right temporal. ((+) = present, (-) = absent symptoms.) * Died. ^ Leigh syndrome.

## References

[1] Vercellino I., Sazanov L.A. (2022). The assembly, regulation and function of the mitochondrial respiratory chain. Nat. Rev. Mol. Cell Biol..

[2] Fernández-Vizarra E., Zeviani M. (2015). Nuclear gene mutations as the cause of mitochondrial complex III deficiency. Front. Genet..

[3] Scaglia F., Towbin J.A., Craigen W.J., Belmont J.W., Smith E.O., Neish S.R., Ware S.M., Hunter J.V., Fernbach S.D., Vladutiu G.D. (2004). Clinical spectrum, morbidity, and mortality in 113 pediatric patients with mitochondrial disease. Pediatrics.

[4] Bansept C., Gaignard P., Lebigot E., Eyer D., Delplancq G., Hoebeke C., Mazodier K., Ledoyen A., Rouzier C., Fragaki K. (2023). UQCRC2-related mitochondrial complex III deficiency, about 7 patients. Mitochondrion.

[5] Gaignard P., Eyer D., Lebigot E., Oliveira C., Therond P., Boutron A., Slama A. (2017). UQCRC2 mutation in a patient with mitochondrial complex III deficiency causing recurrent liver failure, lactic acidosis and hypoglycemia. J. Hum. Genet..

[6] Miyake N., Yano S., Sakai C., Hatakeyama H., Matsushima Y., Shiina M., Watanabe Y., Bartley J., Abdenur J.E., Wang R.Y. (2013). Mitochondrial complex III deficiency caused by a homozygous UQCRC2 mutation presenting with neonatal-onset recurrent metabolic decompensation. Hum. Mutat..

[7] Burska D., Stiburek L., Krizova J., Vanisova M., Martinek V., Sladkova J., Zamecnik J., Honzik T., Zeman J., Hansikova H. (2021). Homozygous missense mutation in UQCRC2 associated with severe encephalomyopathy, mitochondrial complex III assembly defect and activation of mitochondrial protein quality control. Biochim. Biophys. Acta Mol. Basis Dis..

[8] Ogawa E., Fushimi T., Ogawa-Tominaga M., Shimura M., Tajika M., Ichimoto K., Matsunaga A., Tsuruoka T., Ishige M., Fuchigami T. (2020). Mortality of Japanese patients with Leigh syndrome: Effects of age at onset and genetic diagnosis. J. Inherit. Metab. Dis..

[9] Smolen C., Myers S., Girirajan S., Adam M.P., Bick S., Mirzaa G.M., Pagon R.A., Wallace S.E., Amemiya A. (1993). 16p12.2 Recurrent Deletion. GeneReviews(^®^).

[10] Muylle E., Jiang H., Johnsen C., Byeon S.K., Ranatunga W., Garapati K., Zenka R.M., Preston G., Pandey A., Kozicz T. (2022). TRIT1 defect leads to a recognizable phenotype of myoclonic epilepsy, speech delay, strabismus, progressive spasticity, and normal lactate levels. J. Inherit. Metab. Dis..

[11] Rodenburg R.J. (2011). Biochemical diagnosis of mitochondrial disorders. J. Inherit. Metab. Dis..

[12] Ligezka A.N., Budhraja R., Nishiyama Y., Fiesel F.C., Preston G., Edmondson A., Ranatunga W., Van Hove J.L.K., Watzlawik J.O., Springer W. (2023). Interplay of Impaired Cellular Bioenergetics and Autophagy in PMM2-CDG. Genes.

[13] Braissant O., McLin V.A., Cudalbu C. (2013). Ammonia toxicity to the brain. J. Inherit. Metab. Dis..

[14] Hertig D., Felser A., Diserens G., Kurth S., Vermathen P., Nuoffer J.M. (2019). Selective galactose culture condition reveals distinct metabolic signatures in pyruvate dehydrogenase and complex I deficient human skin fibroblasts. Metabolomics.

[15] Gatti D.L., Tzagoloff A. (1990). Structure and function of the mitochondrial bc1 complex. Properties of the complex in temperature-sensitive cor1 mutants. J. Biol. Chem..

[16] Oláhová M., Ceccatelli Berti C., Collier J.J., Alston C.L., Jameson E., Jones S.A., Edwards N., He L., Chinnery P.F., Horvath R. (2019). Molecular genetic investigations identify new clinical phenotypes associated with BCS1L-related mitochondrial disease. Hum. Mol. Genet..

[17] Hikmat O., Isohanni P., Keshavan N., Ferla M.P., Fassone E., Abbott M.A., Bellusci M., Darin N., Dimmock D., Ghezzi D. (2021). Expanding the phenotypic spectrum of BCS1L-related mitochondrial disease. Ann. Clin. Transl. Neurol..

[18] Magner M., Dvorakova V., Tesarova M., Mazurova S., Hansikova H., Zahorec M., Brennerova K., Bzduch V., Spiegel R., Horovitz Y. (2015). TMEM70 deficiency: Long-term outcome of 48 patients. J. Inherit. Metab. Dis..

[19] Mascalchi M., Montomoli M., Guerrini R. (2018). Neuroimaging in mitochondrial disorders. Essays Biochem..

[20] Eichler E.E., Johnson M.E., Alkan C., Tuzun E., Sahinalp C., Misceo D., Archidiacono N., Rocchi M. (2001). Divergent origins and concerted expansion of two segmental duplications on chromosome 16. J. Hered..

[21] Morava E., Rodenburg R., van Essen H.Z., De Vries M., Smeitink J. (2006). Dietary intervention and oxidative phosphorylation capacity. J. Inherit. Metab. Dis..

[22] Danhauser K., Smeitink J.A., Freisinger P., Sperl W., Sabir H., Hadzik B., Mayatepek E., Morava E., Distelmaier F. (2015). Treatment options for lactic acidosis and metabolic crisis in children with mitochondrial disease. J. Inherit. Metab. Dis..

[23] Parikh S., Goldstein A., Karaa A., Koenig M.K., Anselm I., Brunel-Guitton C., Christodoulou J., Cohen B.H., Dimmock D., Enns G.M. (2017). Patient care standards for primary mitochondrial disease: A consensus statement from the Mitochondrial Medicine Society. Genet. Med..

