# Supplementary material for: Reversible Metabolic and Liver Disease in Complex III Deficiency: Novel Variants Expand the Reported UQCRC2-Associated Phenotype
Source: Cells. 2026 Mar 27;15(7):596. doi: 10.3390/cells15070596 (PMC13072357; doi:10.3390/cells15070596)
Supplement: Supplementary file 1 [file cells-15-00596-s001.zip › cells-4090037-supplementary.pdf]

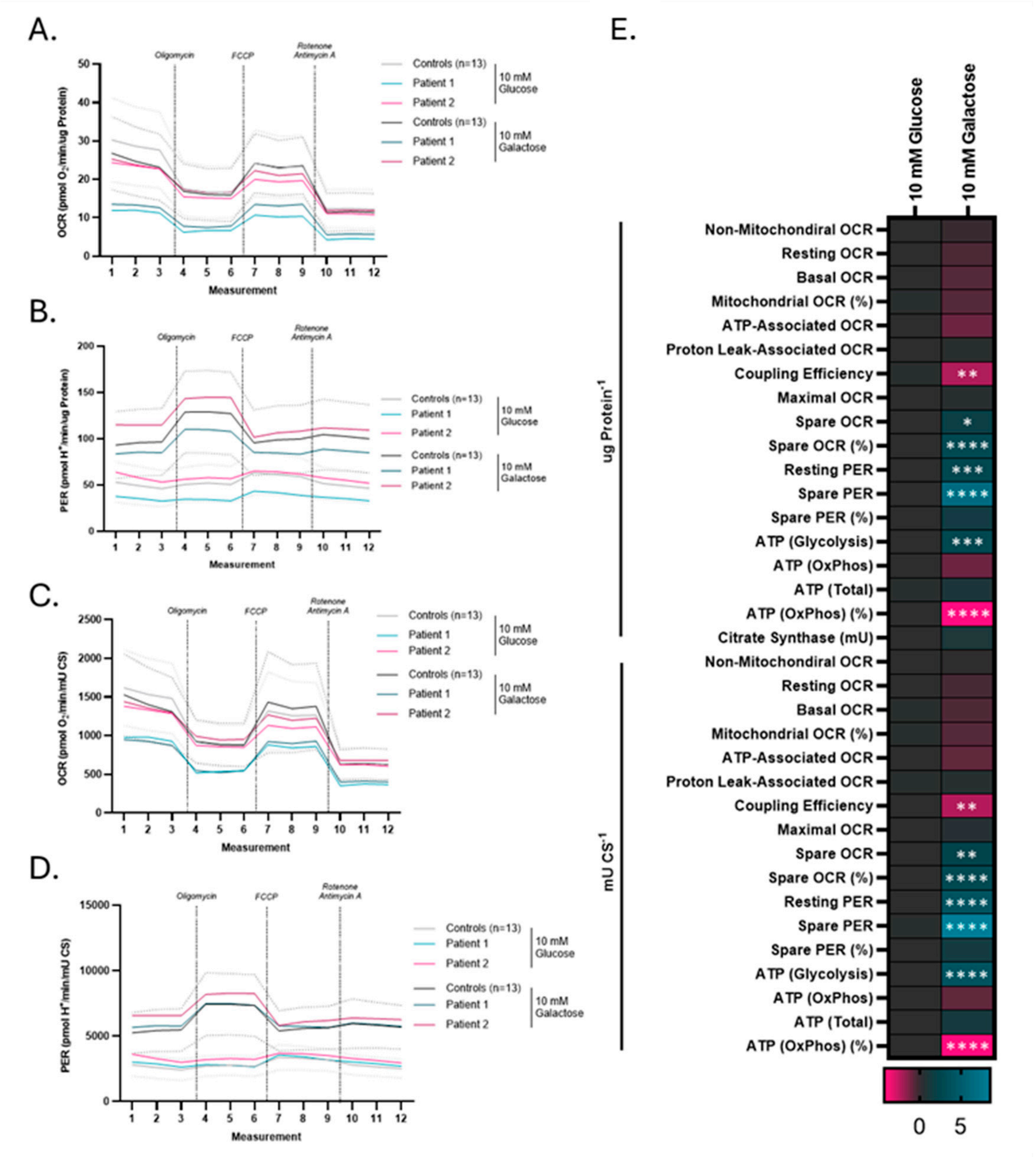


**Figure S1.** Seahorse respirometry in glucose- and galactose-supplemented UQCRC2 and control (n=13) fibroblasts, normalized to both protein concentration and citrate synthase (CS) abundance. A-D) Oxygen consumption rates (OCR) (A,C) and proton efflux rates (PER) (B,D) in *UQCRC2* and control (n=13) fibroblasts supplemented with 10 mM glucose (A,B) or galactose (C,D) over a Seahorse XF Cell Mito Stress Test experiment. E. Heatmap of Seahorse respirometry readouts in control fibroblasts (n=13) supplemented with 10 mM glucose or galactose for 24 hours. Plotted is standard deviations from the mean of the glucose-supplemented controls. Sidak's multiple comparisons test p<0.05 (*), p<0.01 (**), p<0.001 (***), p<0.0001 (****).
